# Flux and Fouling Behavior of Graphene Oxide-Polyphenylsulfone Ultrafiltration Membranes Incorporating ZIF-67/ZIF-8 Fillers

**DOI:** 10.3390/membranes15100289

**Published:** 2025-09-25

**Authors:** Azile Nqombolo, Thollwana Andretta Makhetha, Richard Motlhaletsi Moutloali, Philiswa Nosizo Nomngongo

**Affiliations:** 1 Department of Chemical & Earth Sciences, University of Fort Hare, Alice 5700, South Africa; 2 Department of Chemical Sciences, University of Johannesburg, Doornfontein Campus, P.O. Box 17011, Doornfontein 2028, South Africa; tamakhetha@uj.ac.za (T.A.M.); pnnomngongo@uj.ac.za (P.N.N.); 3 Institute for Nanotechnology and Water Sustainability, College of Science, Engineering and Technology, University of South Africa, Florida Science Campus Florida, Johannesburg 1709, South Africa; moutlrm@unisa.ac.za; 4 Department of Science, Technology and Innovation-National Research Foundation South African Research Chair Initiative (DSTI-NRF SARChI) in Nanotechnology for Water, University of Johannesburg, Doornfontein 2028, South Africa

**Keywords:** metal-organic frameworks, graphene oxide, (ZIF-67/ZIF-8)/GO/PPSU mixed matrix membranes, hydrophilicity, antifouling properties

## Abstract

Wider adoption of membrane technology is hindered by fouling and flux/rejection challenges. Recent practice in mitigating these is to incorporate hydrophilic and porous fillers. Herein the addition of hydrophilic graphene oxide (GO) in conjunction with porous mixed ZIFs (ZIF-67/ZIF-8) crystallites were used as inorganic fillers in the preparation of polyphenylenesulfone (PPSU) ultrafiltration (UF) membranes. The morphology of the resultant composite membranes was assessed using atomic force microscopy (AFM) and scanning electron microscopy (SEM) whilst surface hydrophilicity through water contact angle. The pure water flux (PWF) and membrane permeability were found to increase with increasing filler content. This was attributed to the combined hydrophilicity of GO and porous structure of the ZIF materials because of increasing alternative water pathways in the membrane matrix with increasing filler content. Furthermore, the increase in the ZIF component led to increasing bovine serum albumin (BSA) fouling resistance as demonstrated by increasing fouling recovery ratio (FRR). The dye rejection was due to a combination of electrostatic interaction between the fillers and the dyes as well as size exclusion. The chemical interactions between the ZIFs and the dyes resulted in slightly different rejection profiles for the smaller dyes, the cationic methylene blue being rejected less efficiently than the anionic methyl orange, potentially leading to their separation. The larger anionic dye, Congo red was rejected predominately through size exclusion.

## 1. Introduction

Microfiltration (MF) [1,2], ultrafiltration (UF) [3], nanofiltration (NF) [4] and reverse osmosis (RO) [5] membranes are used in water purification because of their ability to reject bacteria, organic dyes, heavy metals, and salts [3,6]. Polymeric membranes are widely used in water purification, and polymers such as polyacrylonitrile (PAN) [7,8], cellulose acetate (CA) [9] polyvinylidene fluoride (PVDF) [10,11], and polysulfone (PSF) [3], among others have been used in ultrafiltration membranes. Amongst these, PSF is regarded as a good UF material membrane due to its alkaline and acid resistance, good thermal and mechanical stability [1]. However, polysulfone membranes are prone to fouling due to hydrophobic interactions between the solute and the membrane surface. Fouling (both reversible and irreversible) in membranes leads to a decrease in the selectivity of the membrane as well as water flux, and this results in high operational costs [12,13,14,15,16,17].

Polyphenylenesulfone (PPSU) is a relatively new class of polysulfone membrane materials used in UF membranes owing to its resistance to several proteins [18,19,20]. PPSU will have relatively lower electrostatic interactions with negatively charged protein molecules thereby having higher fouling resistance towards proteins/biomolecules compared to widely used PES or PSF [21,22,23], a unique advantage over polyethersulfone (PES) and polysulfone (PSF) which are widely used in membrane technology. When compared to these two sulfone polymers (PSF and PES), PPSU also shows excellent mechanical and thermal stability, resistance to cracking from environmental stress [24]. These attractive properties are however, negated by its relative hydrophobic nature compared to both PES or PSF, making its membranes prone to bio and organic fouling as these types of pollutants can easily adhere to its membrane surface and lead to severe fouling [1,25]. In order to overcome these setbacks, hydrophobic membranes are modified by incorporating hydrophilic and porous/selective fillers such as graphene oxide and metal organic frameworks (MOF), specifically the sub-clusters of MOFs such as zeolitic imidazole frameworks (ZIFs) [26]. The incorporation of these fillers can be done using different methods, such as grafting [7,27], blending [28,29], and coating [30,31,32,33]. It has been reported that incorporating adsorptive/selective nanomaterials and hydrophilic materials decreases membrane fouling thereby reducing the operational costs [34,35].

Recent work done by different researchers [35,36,37] showed a decrease in surface roughness, which played a crucial role in fouling resistance. It was also reported that the composite membranes with ZIFs and graphene oxide showed an increase in hydrophilicity, resulting in enhanced water flux in comparison to the pristine PES polymer used [36,38]. Interestingly, high flux recovery values were also reported, i.e., ~90% and >95% [36,37,38].

Currently, researchers are working on the use of ZIF-67 and ZIF-67@GO hybrid for wastewater treatment since ZIF-67 exhibits similar properties to those of ZIF-8 [39,40]. Cheong et al. [41] reported high adsorption capacity (80%) and higher photodegradation efficiency (90.5%) of methylene blue using ZIF-67@GO/PAN membranes. Furthermore, new developments have been made on the use of hybrid ZIFs for wastewater treatment [42]. Li et al. reported high removal efficiency (more than 85%) of pesticides using Fe_3_O_4_-ZIF-8@ZIF-67. Abdelhammed et al. [43] described the antibacterial studies of ZIF-67@ZIF-8@MIL-125-NH_2_ on *S. aureus* as being higher in comparison to that of MOFs used separately. The increased use of the mixed ZIF-8/ZIF-67 composite is due to the fact that it exhibits enhanced stability. For example, ZIF-8 is reported to be easily degraded in the presence of oxygen/moisture [44,45] whilst ZIF-67 is unstable at elevated temperatures [46]. Recent studies have shown that combining these two led to an overall more stable ZIF with respect to both oxygen, moisture and elevated temperatures [47,48]. In addition, the mixed ZIF possessed high adsorption and catalytic properties compared to the components separately [49]. Furthermore, the mixed ZIF-8/ZIF-67 framework offers a promising application in the separation of similarly structured molecules [50]. Incorporating such highly efficient molecular sieving components into polymeric membranes to explore synergies inherent in such combinations is attractive for solute separation in wastewater treatment. According to our knowledge, there are no reports of the incorporation of mixed ZIF-8/ZIF-67 framework structures into the PPSU UF membranes for dye rejection. This knowledge gap prompted us to explore the influence of the mixed ZIF-8/ZIF-67 on the performance of PPSU UF membranes in dye rejection and separation applications.

Herein, we report on UF membranes incorporating (ZIF-67/ZIF-8)/GO hybrid for simulated textile wastewater treatment. It was envisaged that the differences in surface and pore structural properties of ZIF-8 compared to ZIF-67 would lead to a selective dye rejection mechanism when used on cationic and anionic dyes. In addition, the additional pathways through the porous ZIF structure, combined with the hydrophilic properties of GO will result in membranes possessing high water flux. Most importantly, they might present improved fouling resistance toward proteins due to decreased attaching sites through reduced surface roughness and enhanced surface hydrophilicity.

## 2. Experimental

### 2.1. Materials 

All solvents and chemicals were used as purchased. Ethanol, methanol, polyvinylpyrrolidone (PVP 40,000 g·mol^−1^), N-methyl-2-pyrrolidone (NMP), polyphenylsulfone (PPSU 400.45 g/mol) graphite, sodium nitrate, sulphuric acid, potassium permanganate, phosphoric acid, hydrochloric acid, ethanol, Congo red, methylene blue, methyl orange, Bovine serum albumin (BSA), ammonium solution and hydrogen peroxide were all purchased from Merck (St. Louis, MO, USA) and used as obtained. The instrumental grade nitrogen gas was used in the dead-end cell from Afrox (Johannesburg, South Africa).

### 2.2. Synthesis of ZIF-67/ZIF-8 Filler

The ZIF-67/ZIF-8 was prepared according to our previous study [50] while GO was synthesised according to the reported method [51].

### 2.3. Preparation of PPSU Membranes

Membranes were prepared by the phase invasion method where GO (0.1 wt.%), PVP (2 wt.%) and ZIF-67-/ZIF-8 were dispersed in NMP and sonicated to mitigate aggregation of the composite fillers [34] followed by the addition of PPSU (16 wt.%) as seen in Table 1. The mixture was stirred at 60 °C for 12 h. To remove bubbles, a desiccator was used to degas the casting solution prior to casting the UF membranes. The solution was cast on a glass plate using a casting knife (200 μm) and then immersed in a deionized water coagulation bath (25 °C). The membranes were left in deionized water for 24 h to completely remove NMP solvent. The mixed matrix membranes were cut and dried for characterization while some were put in water and stored in the fridge to be later used for performance.

### 2.4. Instrumentation

#### 2.4.1. Atomic Force Microscopy (AFM) and Scanning Electron Microscopy (SEM)

The prepared membranes were cut from different locations to have different samples for cross-section using TESCAN VEGA3 scanning electron microscopy (SEM) (TESCA, Brno, Czech Republic). The membrane samples for both cross-section and surface were coated with carbon prior to analysis. Atomic force microscopy (AFM) (model Multimode-V, Veeco Bruker, Ulm, Germany) nanoscale 1v was used to measure the surface roughness of the PPSU membranes.

#### 2.4.2. Contact Angle and Flux

A contact angle goniometer (G10, KRUSS, Hamburg, Germany) was used to check the hydrophilicity or hydrophobicity of each membrane. Deionised was used to perform this by placing a drop on the membrane surface and the images were captured in 30 s intervals. The values of the contact angles were depleted from the average of the 10 drops at different positions of the membrane surface.

The required piece of the membrane with an area of 12.6 cm^2^ was cut for loading in the dead-end cell. In order to adjust the pressure applied in the membrane inside the cell, compressed nitrogen gas was used. Each membrane was compacted. at a pressure of 200 kPa for 30 min before evaluation. Water flux (*J_flux_*) was then noted at six pressures from 50–180 kPa at 5 min intervals, and then the water pure flux was calculated using Equation (1) as follows:

(1) $$J_{flux}=\frac{Q}{A.t}$$

where *t* is the permeation time (h), *J_flux_* is the permeate flux of the membrane (L·m^−2^h^−1^), *A* is the effective area (m^2^) and *Q* is the volume of water permeate (L).

#### 2.4.3. Rejection Studies

Three different dyes, Congo red, methyl orange and methylene blue were used to assess the solute rejection of the membranes. The dye solution in the dead-end cell was stirred during filtration to mitigate against concentration-effects during the tests. The concentration of the dyes was 100 mg·L^−1^ and about 15 mL of permeate was collected in triplicates. Ultraviolet spectrophotometer UV-2450 (Shimadzu, Kyoto, Japan) was used to measure the concentration of dyes in feed and permeate solutions. The spectral window was between 190–800 nm and the rejection ratio was determined using Equation (2):

(2) $$\begin{array}{l} R(\%)=(1-\frac{C_{p}}{C_{f}})\times 100 \end{array}$$

#### 2.4.4. Fouling Studies

Bovine serum albumin (BSA, 1000 mg·L^−1^) protein with a MW of 69 kDa was used as a foulant for rejection analysis. Firstly, the membranes were subjected to pure water flux for 1 h (*J_w_*_1_) followed by filtration of the BSA solution for 1 h to obtain *J_P_*. The fouled membrane was rinsed and soaked in deionized water for 1 h. The flux of the clean membrane was measured (*J_w_*_2_). The resultant total fouling and flux recovery ratio were calculated from Equation (3). Other antifouling properties i.e., total fouling ($R_{t}$), reversible fouling ratio ($R_{r}$), and irreversible fouling ratio ($R_{ir}$) were assessed using Equations (4), (5) and (6) respectively [51].

(3) $$FRR\left(\%\right)=\frac{J_{w2}}{J_{w1}}\;\times 100$$

(4) $$R_{t}\left(\%\right)=\left(1-\frac{J_{p}}{J_{w1}}\right)\times 100$$

(5) $$R_{r}\;\left(\%\right)=\left(\frac{J_{w2}-J_{p}}{J_{w1}}\right)\times 100$$

(6) $$R_{ir}\;\left(\%\right)=\left(\frac{J_{w1}-J_{w2}}{J_{w1}}\right)\times 100=R_{t}-R_{r}$$

## 3. Results and Discussion

### 3.1. Scanning Electron Microscopy (SEM)

**Figure 1:**
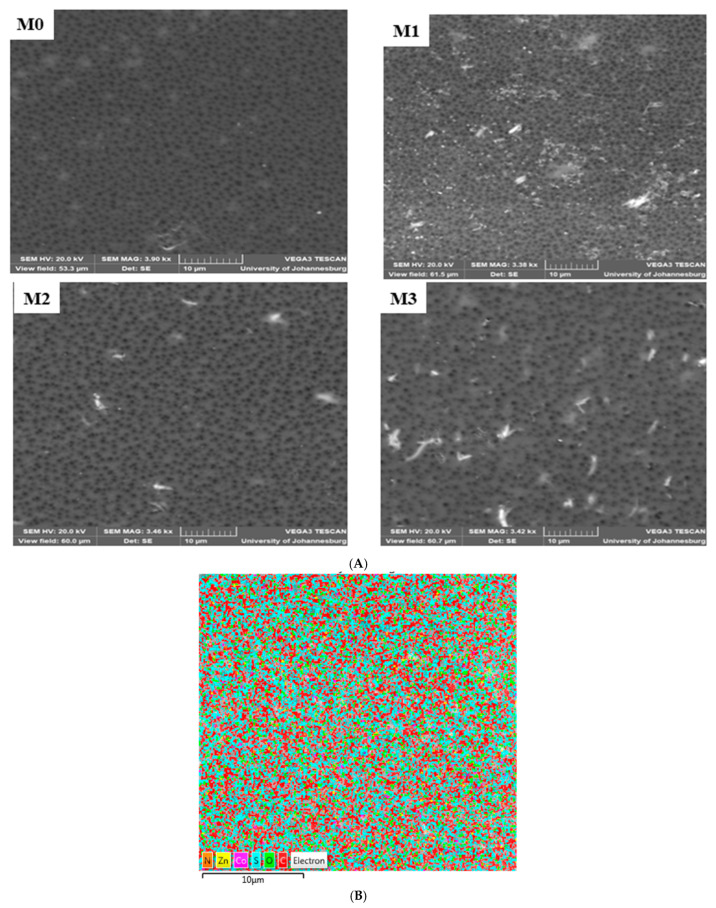
(**A**): SEM images (surface) of M0 (PPSU), M1 (ZIF-67/ZIF-8)_0.3_/GO/PPSU), M2 ((ZIF-67/ZIF-8)_0.5_/GO/PPSU) and M3: (ZIF-67/ZIF-8)_0.7_/GO/PPSU) membranes. (**B**): EDS mapping for M3.

**Figure 2:**
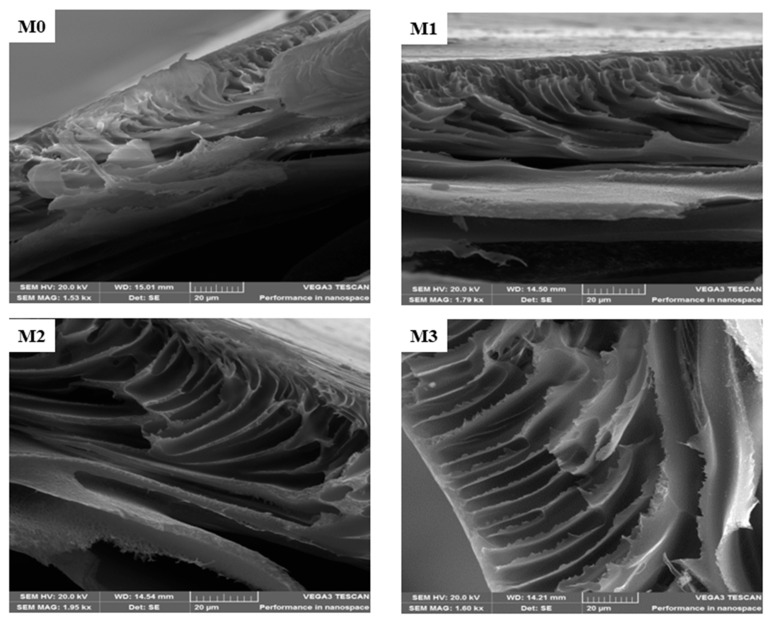
Cross-section SEM micrographsof M0 (PPSU), M1 ((ZIF-67/ZIF-8)_0.3_/GO/PPSU), M2 ((ZIF-67/ZIF-8)_0.5_/GO/PPSU) and M3 (ZIF-67/ZIF-8)_0.7_/GO/PPSU) membranes.

The surface morphology of the mixed matrix membranes (MMM) is shown in Figure 1A. The pristine PPSU exhibits uniformly distributed relatively smaller pores compared to the mixed matrix membranes. The SEM (surface) of mixed matrix membranes showed no evidence of agglomeration of ZIF or GO on them. Further evidence for this is the absence of brittleness and cracks in the membranes [52]. The (ZIF-67/ZIF-8)_0.7_/GO/PPSU membrane showed large pores compared to other membranes; and this is attributed to the increase in the porous ZIF-67/ZIF-8 content loading. The fast non-solvent to solvent exchange during phase invasion process resulted in an increase in membrane pores. A comparison of the cross-sections of the pristine PPSU and mixed matrix membranes is presented in Figure 2. This revealed that the membranes exhibit a dense selective top layer and a finger-like sub-layer. The mixed matrix membrane showed wider pores relative to the base PPSU membrane indicative of the accelerated exchange between non-solvent (water) and solvent (NMP) during membrane preparation owing to the porous ZIF-67/ZIF-8 material and plenty of hydrophilic groups from GO. It is envisaged that this will in turn enhance the filtration efficiency of the mixed matrix membranes. EDS mapping (Figure 1B) was done to confirm the elemental composition in the MMM. All the expected elements (Zn, Co, N and C) from the fillers and the polymer (S, O and C) were observed.

### 3.2. Atomic Force Microscopy (AFM)

**Figure 3:**
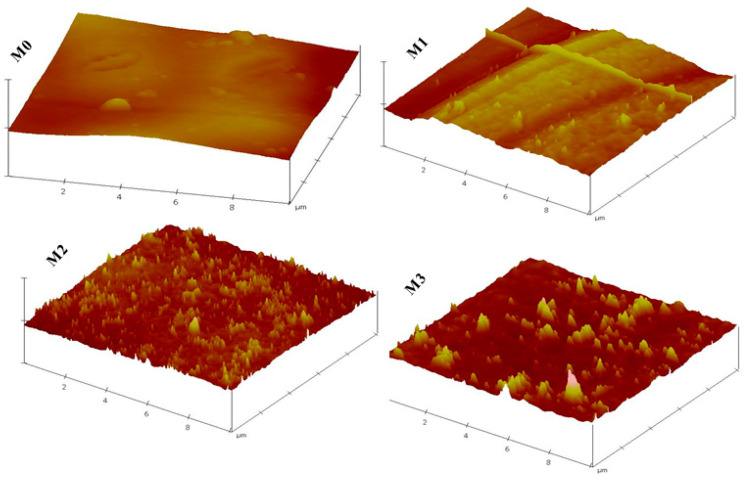
AFM images of M0 (PPSU), M1 (ZIF-67/ZIF-8)_0.3_/GO/PPSU), M2 (ZIF-67/ZIF-8)_0.5_/GO/PPSU) and M3 (ZIF-67/ZIF-8)_0.7_/GO/PPSU) membranes.

Further surface analyses of the membranes were achieved by AFM characterisation (Figure 3) from which surface roughness parameters were determined. Prevailing knowledge indicates that membranes with low surface roughness enhance surface antifouling properties by decreasing the adsorption of the pollutant on the membrane surface [52]. However, in this study, an increase in surface roughness was observed in Figure 3 and Table 2, indicating that the filler material was not only integrated into the PPSU membrane but also onto the surface of the membrane as observed in SEM images in Figure 1. For instance, the Ra value increased from 15.13 nm for the pristine membrane whilst those for the mixed matrix membranes ranged from 26.49 (0.3 wt.%) to 52.59 nm (0.7 wt.%). The increase in surface roughness could be due to the aggregation of the ZIFs/GO on the surface of the MMM [53]. Previous studies suggest that a membrane with increasing surface roughness due to the addition/incorporation of the nanomaterials results in a composite membrane that has a more effective area to contact water through the filtration process, thereby increasing water permeability [53,54].

### 3.3. Contact Angle Analysis

**Figure 4:**
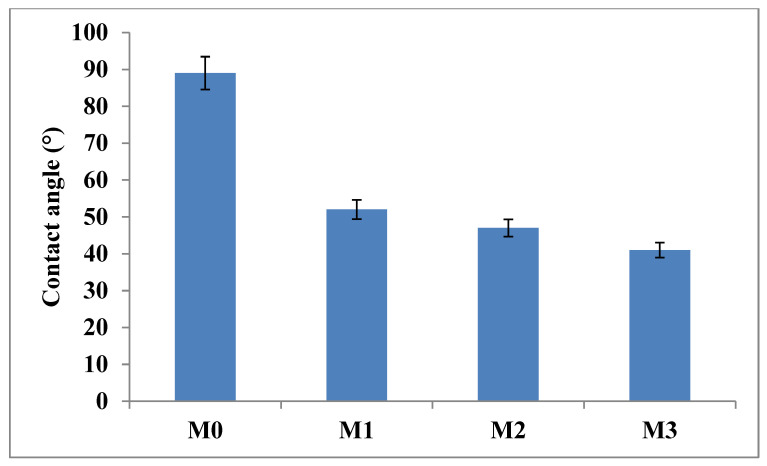
Contact angle measurements for M0 (PPSU), M1 ((ZIF-67/ZIF-8)_0.3_/GO/PPSU), M2 ((ZIF-67/ZIF-8)_0.5_/GO/PPSU) and M3 ((ZIF-67/ZIF-8)_0.7_/GO/PPSU) membranes.

Membrane hydrophilicity was determined using contact angle analysis; membranes with low contact angle have high hydrophilicity while membranes with high contact angle are said to be hydrophobic [17,55]. The contact angle value of the pristine PPSU membrane (Figure 4) was 89° indicative of its relative hydrophobicity. The incorporation of inorganic fillers (GO and ZIF-67/ZIF-8) led to a decrease in the contact angle of the mixed matrix membranes down to 41°. The GO and ZIF were well dispersed on the membrane surface hence the increase in hydrophilicity, due to the oxygenated functional groups of GO. The porous ZIF-67/ZIF-8 interacts with GO hence a slight increase was observed when ZIF-67/ZIF-8 content was increased. The increase in ZIF-67/ZIF-8 content/loading at constant GO loading (0.1 wt.%) was not dramatic on the CA, indicating that the initial drop in CA was predominantly due to the GO component and hence on the membrane hydrophilic character. Having hydrophilic fillers on the membrane will form a hydration layer which will then repel the hydrophobic foulants during filtration [51]. Subsequently, this will enhance the pure water flux and the antifouling properties of the MMM., similar observation has been reported on ZIF fillers previously [34,56,57].

### 3.4. Membrane Water Flux and Permeability

**Figure 5:**
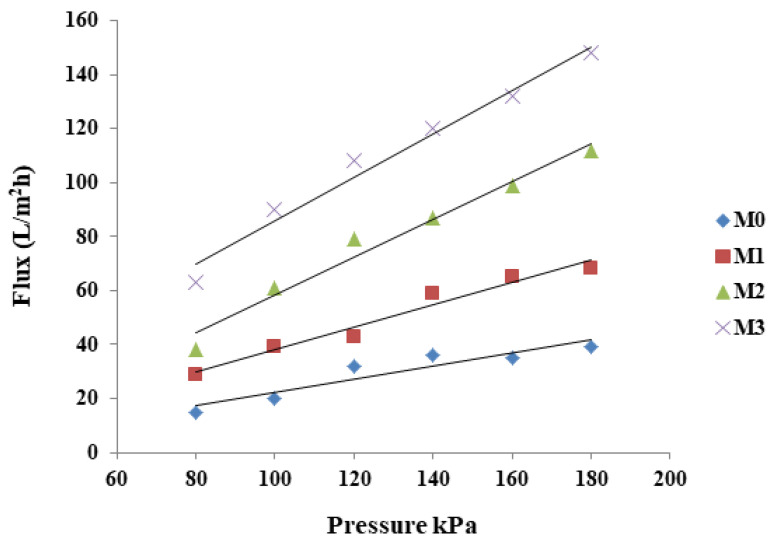
Pure water flux of M0 (PPSU), M1 ((ZIF-67/ZIF-8)_0.3_/GO/PPSU, M2 ((ZIF-67/ZIF-8)_0.5_/GO/PPSU) and M3 ((ZIF-67/ZIF-8)_0.7_/GO/PPSU) membranes.

Pure water flux of the MMM at different pressures (80–190 kPa) was assessed in a dead-end cell and the data are presented in Figure 5. Pure PPSU membrane showed the lowest water flux, which progressively increased with increasing inorganic filler (ZIF-67/ZIF-8) content in the composite membranes. The introduction of the porous ZIFs creates new/additional pathways for water passage [52] which is also reflected in the membrane permeability at increasing pressures (Figure 5). The addition of GO improves the surface hydrophilicity that helps to absorb water thereby facilitating water penetration through the membranes [55,58]. Thus, the combination of ZIF and GO in the mixed matrix membranes led to a relatively high-water flux than the baseline PPSU membrane.

### 3.5. Water Uptake and Porosity of the Prepared Membranes

The porosity and water uptake of PPSU membranes were studied according to literature methods [59]. The membranes were cut into small round pieces area (12.6 cm^2^) and soaked in water overnight. The membranes were then weighed to obtain the mass of the wet membrane (*W_w_*) and dried at 60 °C for 10 h and weighed again to get the mass of the dry membrane (*W_d_*). The water update was calculated using Equation (7) while membrane porosity was determined using Equation (8) [51].

(7) $$\%\;Water\;uptake=\frac{W_{w}-W_{d}}{W_{d}}\;\times \;100$$

(8) $$\varepsilon (\%)=\frac{W_{w}-W_{d}}{\rho Al}$$

where *ρ* is the density of deionised water (0.998 g·cm^−3^), ε denotes the porosity (%), *A* is the membrane effective area (m^2^), *l* is the membrane thickness (m). *W_d_* and *W_w_* are dry and wet membranes respectively. The porosity and water uptake of the membranes are captured in Table 3. The observed water uptake was 44.5%, 62.4%, 76%, and 79.3% for M0, M1, M2, and M3 respectively. The increased membrane porosity and water uptake tracked the increasing ZIF content, demonstrating that the porous ZIF materials enhance water uptake in the MMM. The combination of hydrophilic sites in the membrane surface and porosity determines the water uptake ability [60].

Additionally, hydraulic permeability (slopes Figure 5) was determined by measuring water flux at different applied pressures. Increasing ZIF-67/ZIF-8 loading resulted in an increase in the permeability of the membranes. For instance, the permeability of M3 was more than three times that of M0. The observations that increasing the content of ZIF-67/ZIF-8 influenced membrane porosity are in line with reported trends [61].

### 3.6. Dye Rejection

**Figure 6:**
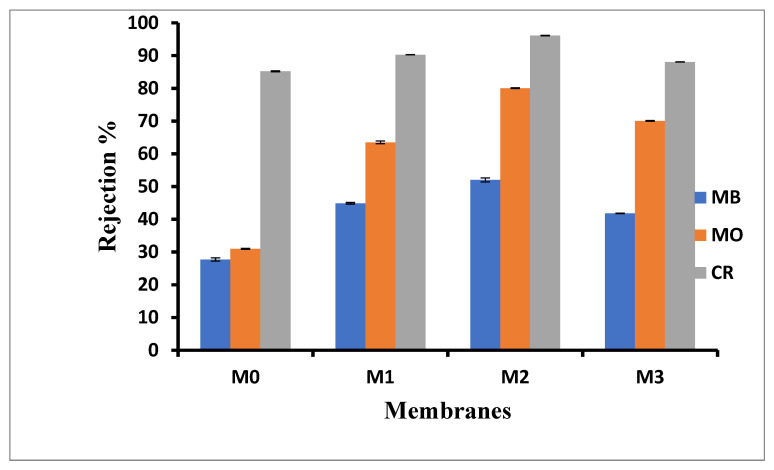
Membrane performance in dye rejection for M0 (PPSU), M1 ((ZIF-67/ZIF-8)_0.3_/GO/PPSU), M2 ((ZIF-67/ZIF-8)_0.5_/GO/PPSU) and M3 ((ZIF-67/ZIF-8)_0.7_/GO/PPSU) membranes.

Figure 6 shows rejection of Congo red (CR, 696.67 g·mol^−1^) methyl orange (MO, 327.33 g·mol^−1^) and methylene blue (MB, 319.85 g·mol^−1^) as representative organic dyes, with both MO and CR being hydrophobic anionic dyes whilst MB, a hydrophilic cationic dye. The CR rejection in the current study was to confirm that the membranes were indeed UF membranes; a rejection of 80% and above is indicative of this type of membrane [51]. All membranes showed rejections above this threshold, with the pure PPSU membrane having 85% rejection for CR dye. The CR rejection increased upon the addition of inorganic fillers for M1 to M3. There was a slight decrease in rejection for M3 however, which is attributed to its relatively larger pores (Figure 1). As previously reported, the rejection for CR is mainly a size exclusion effect [51]. For the smaller organic dyes, the rejection of MB was lower than MO. The rejection of MO increased slightly with an increasing ZIF-67/ZIF-8 loading up to M2 decreasing for M3 due to its relatively larger pore sizes. The same trend was observed for MB; in all the cases, its rejection was about 10% lower than that observed for MO. It must be noted that the rejection for both MO and MB was higher than that observed for baseline PPSU membranes, indicative of additional interactions that the ZIF/GO is having with the dye molecules. This is in line with previous observations that MB interacted with ZIF-67 negatively (electrostatic repulsion) whilst MO interacted with ZIF-67 positively (electrostatic attraction) [62]. Thus, the passage of MO is slowed down due to these attractions, which are not the same for MB and hence the differences in rejections observed. Such interactions were reported previously for the smaller molecular weight MB and MO on ZIF-8 as well [51,55,63]. In addition, MOFs have been used as adsorption materials due to specific interaction with contaminants in water [20]. This was confirmed through the observed increase in dye rejection with increasing ZIF content in the mixed matrix membranes. It is thus postulated that, in addition to the sieving mechanism associated with relative pore sizes of the mixed matrix membranes, additional interaction between the ZIF fillers and the charged dyes influenced the observed increase in rejection for the smaller dyes.

### 3.7. Membrane Fouling

**Figure 7:**
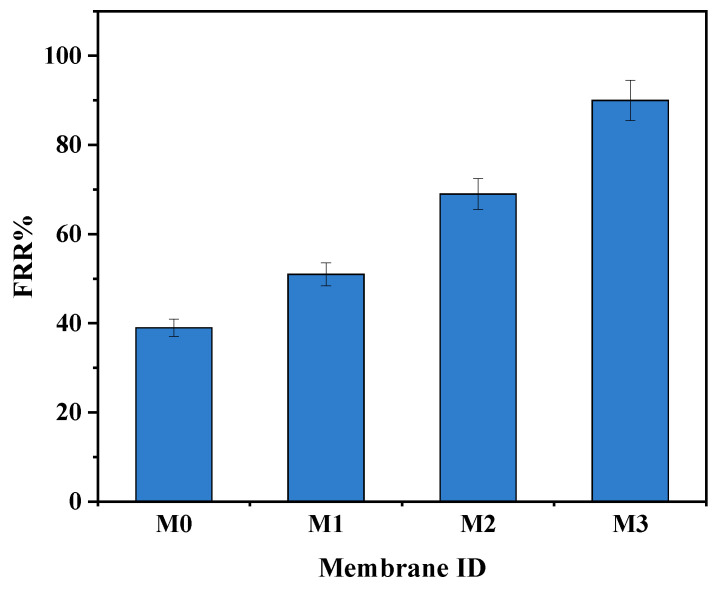
FRR for M0 (PPSU), M1 ((ZIF-67/ZIF-8)_0.3_/GO/PPSU), M2 ((ZIF-67/ZIF-8)_0.5_/GO/PPSU) and M3 ((ZIF-67/ZIF-8)_0.7_/GO/PPSU) membranes.

**Figure 8:**
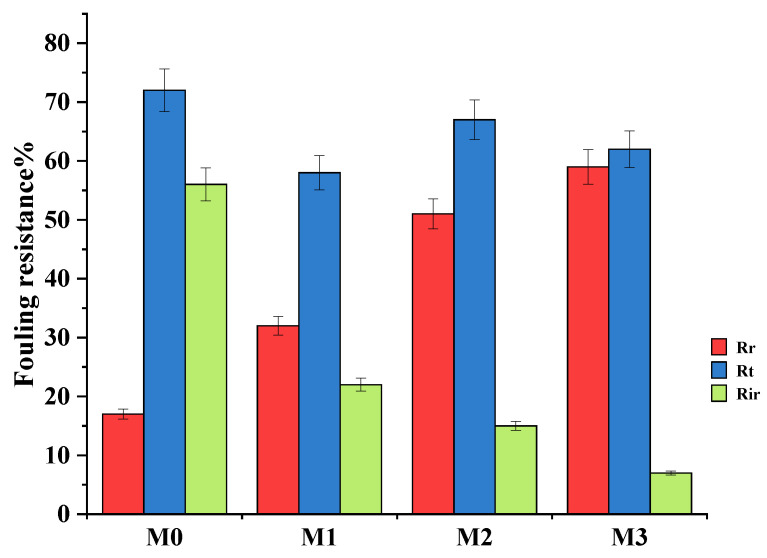
Fouling resistance of the prepared membrane.

Bovine serum albumin (BSA, 100 mg·L^−1^) in the feed solution was used as a fouling agent. The increase in BSA rejection is confirmation that the high pure water flux was not due to defects or cracks of the surface membranes (Figure 7). After washing the fouled membranes, the pure PPSU membrane had a higher flux decline compared to mixed matrix membranes attributed to its relatively higher surface hydrophobicity. The 0.7 wt.% showed the highest fouling recovery ratios indicative of its high antifouling characteristics attributed to its highest inorganic filler content. The increased hydrophilicity at higher filler content (Figure 4) facilitated adsorption of water molecules onto the membrane surface forming a tight hydration layer that delays/prevents attachment of the foulant thereby increasing antifouling characteristics of mixed matrix membranes. Therefore, the influence of the ZIF-67/ZIF-8 on PPSU membrane antifouling performance was demonstrated. The high flux recovery ratio (FRR) was the reflection of the contribution to higher surface hydrophilicity of mixed matrix membranes (Figure 7) that controls the adsorption of pollutants in line with previous reports [23]. The increase in membrane hydrophilicity due to the mixed ZIF additives (Figure 4, Table 2) led to increased FRR in line with prior reports [20]. For the pure PPSU, the FRR values were low which is indicative of serious membrane fouling. The BSA molecules aggregated or were trapped in the walls of the pure PPSU membrane, hence a fouling layer was generated. It is difficult to completely clean the fouling layer due to the strong hydrophobic interaction between BSA and PPSU material. Upon addition of (ZIF-67/ZIF-8)/GO, the values of mixed matrix membranes increased and at 0.7 wt.% of ZIF-67/ZIF-8, about 90% FRR (Figure 7) was observed, which is indicative of the favourable antifouling ability of mixed matrix membranes [17, 19]. Total fouling (*R_t_*), irreversible fouling (*R_ir_*) and reversible fouling (*R_r_*), were derived to further understand fouling properties of the membranes, and these results are shown in Figure 8. The total fouling of the bare PPSU membrane was higher compared to that of mixed matrix membranes; the *R_ir_* contributed to this high *R_t,_* which occurred through adsorption of BSA onto the surface of the PPSU membrane and pores. The results for the membranes (M1–M3) indicated that the *R_ir_* fouling decreased with the addition of ZIF-67/ZIF-8/GO fillers. The GO added into the membrane enhanced the hydrophilicity of PPSU membranes hence the antifouling properties were improved upon the addition of the fillers.

### 3.8. Comparative Studies

The prepared MMM was compared with previous studies that used either MOF or GO composite membranes. Table 4 shows the performance of other reported membranes for various dye rejections. The ZIF/GO PPSU membrane has comparable results with those reported in the literature. Even though the dye rejection was comparable with previous studies, the increase in antifouling properties and high-water permeability was observed in the mixed matrix membrane.

**Table 4:** Comparative studies of GO-based membranes.

Membrane	Analyte & Performance (%)	References
Cu(tpa)@GO/PES composite membranes	CR: 80% MO: 50% MB: 20%	[51]
Ag@HPEI@GO/PES	MB: 90%	[64]
c-CNT@GO composite membranes	CR: 98.7 MB: 94.1	[65]
GO/MoS2 composites membranes	CR: 99.8% MB: 97.6%	[66]
ZIF-67/ZIF-8/GO PPSU	MB: 52% CR: 96% MO: 80%	This study

## 4. Conclusions

The prepared ZIF-67/ZIF-8/GO PPSU mixed matrix membranes showed promising performance for dye filtration application. The observed decrease in contact angle is indicative of increasing hydrophilic character of the PPSU membranes, attributed to the combined influence of mixed ZIF and GO, which contributed positively to both water flux and fouling behaviour. Thus, the pure water flux increased slightly by increasing the content of ZIF-67/ZIF-8 in the casting solution of each membrane, accompanied by a concomitant increase in membrane permeability. In addition, BSA fouling studies showed that increasing the ZIF-67/ZIF-8 content led to increased resistance to protein fouling as evidenced by progressively increasing FRR. (ZIF-67/ZIF-8)_0.7%_/GO/PPSU showed ca. 90% flux recovery ratio, which is good for membrane reuse and increases its application and life span, thereby decreasing cost and maintenance as membrane cleaning is done using deionized water through the backwash process. The combination of GO and ZIF-67/ZIF-8 fillers resulted in increased antifouling and water flux properties of the resultant composite membranes. The dye rejection was due to the size exclusion and the electrostatic interaction between the filler and the dyes. Congo red with a large molecular weight was rejected more through size exclusion and electrostatic interactions. For the smaller dyes (MB and MO), the rejection was slightly different; the cationic methylene blue showed less rejection compared to the anionic methyl orange, which reflected the differences in how these two dyes interacted with the mixed ZIF additives. For future studies, the rejection of the foulant should be added to compare it with the dye rejection and see which pollutants will show better performance. In addition, the variation of GO while keeping the ZIF content constant should be studied to see if the increase of GO will result in high performance compared to varying the ZIF loading onto the matrix of the membrane. The membrane could be used for longer times to investigate its durability and the stability of the fillers over time.

## Figures and Tables

**Table 1:** Membrane casting solution [17].

Membrane ID	PPSU wt.%	GO wt.%	ZIF-67/ZIF-8 wt.%	PVP wt.%	NMP wt.%
M0	16	0.0	0.0	2	82.0
M1	16	0.1	0.3	2	81.6
M2	16	0.1	0.5	2	81.4
M3	16	0.1	0.7	2	81.2

**Table 2:** AFM results of the prepared membranes [17].

Membranes	R_q_ (nm)	R_a_ (nm)
M0	18.72	15.13
M1	23.76	26.49
M2	43.36	40.98
M3	64.8	52.59

**Table 3:** Effect of mixed matrix membranes on some characterization parameters [17].

Membranes	Permeability (L·m^−2^h^−1^kPa^−1^)	Water Uptake (%)	Porosity (%)
M0	0.241	44.5	76.91
M1	0.412	62.4	85.03
M2	0.7029	76	87.34
M3	0.8043	79	96.13

## Data Availability

The original contributions presented in this study are included in the article. Further inquiries can be directed to the corresponding author.
